# Policing Cancer: Vitamin D Arrests the Cell Cycle

**DOI:** 10.3390/ijms21239296

**Published:** 2020-12-06

**Authors:** Sachin Bhoora, Rivak Punchoo

**Affiliations:** 1 Department of Chemical Pathology, Faculty of Health Sciences, University of Pretoria, Pretoria 0083, Gauteng, South Africa; u15104533@up.ac.za; 2 National Health Laboratory Service, Tshwane Academic Division, Pretoria 0083, South Africa

**Keywords:** cell proliferation, cell cycle, cyclin-dependent kinase, cyclin-dependent kinase inhibitor, vitamin D, calcitriol, anti-proliferation, cancer

## Abstract

Vitamin D is a steroid hormone crucial for bone mineral metabolism. In addition, vitamin D has pleiotropic actions in the body, including anti-cancer actions. These anti-cancer properties observed within in vitro studies frequently report the reduction of cell proliferation by interruption of the cell cycle by the direct alteration of cell cycle regulators which induce cell cycle arrest. The most recurrent reported mode of cell cycle arrest by vitamin D is at the G1/G0 phase of the cell cycle. This arrest is mediated by p21 and p27 upregulation, which results in suppression of cyclin D and E activity which leads to G1/G0 arrest. In addition, vitamin D treatments within in vitro cell lines have observed a reduced C-MYC expression and increased retinoblastoma protein levels that also result in G1/G0 arrest. In contrast, G2/M arrest is reported rarely within in vitro studies, and the mechanisms of this arrest are poorly described. Although the relationship of epigenetics on vitamin D metabolism is acknowledged, studies exploring a direct relationship to cell cycle perturbation is limited. In this review, we examine in vitro evidence of vitamin D and vitamin D metabolites directly influencing cell cycle regulators and inducing cell cycle arrest in cancer cell lines.

## 1. Introduction

Cancer can be described as a heterogeneous disease characterized by uncontrolled cell growth, invasion, and spread from the original site of the disease to several other sites in the body (metastasis) [1]. Cancer is a major cause of death and a barrier to increasing life expectancy in every country of the world in the 21st century, and this is a major global health burden [2]. The need to develop efficient treatments to combat this deadly disease is crucial with a multitude of avenues currently being investigated.

Over the last four decades, observational, pre-clinical, and clinical studies have demonstrated the potential anti-cancer role of vitamin D [3,4]. Almost four decades ago, epidemiological data on sunlight (UVB) exposure and low latitude—both factors that result in higher vitamin D production—showed a lower incidence in colorectal cancer [5]. At the same time, Colston et al. [6] demonstrated reduced cell proliferation of melanoma cell cultures treated with fully activated endogenous vitamin D hormone, 1,25(OH)_2_D_3_. Since then, numerous pre-clinical studies on the anti-cancer actions of vitamin D metabolites have shown promising results [3,7]. However, the mechanisms of anti-cancer properties of vitamin D metabolites remain to be fully elucidated. The current data suggest that a common mode of anti-cancer action of vitamin D and its metabolites is the reduction of cell proliferation by the interruption of the cell cycle. In addition, other mechanisms which have been reported include the promotion of cell death, in particular, apoptosis; and inhibition of tumor metastases and angiogenesis [3,4,8,9,10]. Here we will review the evidence from in vitro studies that investigate vitamin D and vitamin D metabolites on the cell cycle in cancer cell lines.

## 2. An Overview of Vitamin D Metabolism, Signaling and Functions in the Human Body

### 2.1. Vitamin D Metabolism

Vitamin D is a steroid hormone that regulates calcium and phosphate homeostasis. Vitamin D is endogenously produced in the skin where ultraviolet B radiation from sunlight converts 7-dehydrocholesterol to cholecalciferol (vitamin D3) [11]. Alternative sources include vitamin D fortified foods and pharmaceutical supplementation. Irrespective of its source, vitamin D precursors undergo two sequential hydroxylations to form the most active compound. Firstly, vitamin D is hydroxylated by the 25-hydroxylase enzyme (CYP2R1) in the liver to form 25-hydroxyvitamin D, and secondly, 25-hydroxyvitamin D is converted to the active compound by 1α-hydroxylase (CYP27B1) in the kidney to form 1,25-dihydroxyvitamin D (calcitriol) [12]. Both calcitriol and calcidiol are inactivated by renal 24-hydroxylase (CYP24A1) which forms an inactive water-soluble metabolite that is excreted in the bile [12]. The vitamin D metabolizing system (VDMS) consisting of activating (CYP2R1, CYP27B1) and inactivating (CYP24A1) vitamin D enzymes, and expression of vitamin D receptor (VDR) has also been described in selective tissue types, and allows for a narrow homeostatic control of vitamin D metabolism related to cell growth and metabolism at an autocrine/paracrine level.

### 2.2. Vitamin D Intracellular Signalling

Activated vitamin D mediates its actions by binding to the vitamin D receptor (VDR) in the cytoplasm of the target cell [11]. Once bound to the ligand, VDR couples to retinoid X receptor (RXR), and this trimeric complex then enters the nucleus [11]. The complex binds to vitamin D response elements (VDREs) within the nucleus. These are short 15 base pair DNA sequences often comprised of two directly repeated consensus AGGTCA sequences and are commonly located within a three kilobase region of the promoters of target genes [13].

### 2.3. Vitamin D’s Function in the Human Body

Vitamin D has numerous extra-skeletal actions. To date, VDR expression has been reported in healthy and cancerous extra-skeletal target tissue. The actions of calcitriol may directly or indirectly regulate as much as 5% of the human genome [14,15]. The literature best describes the function of vitamin D and its metabolites on optimal bone and mineral metabolism. However; the investigation of the extra-skeletal axis by calcitriol has expanded in the last two decades as evidenced by pre-clinical and clinical studies [3,4]. The growth-regulating actions of 1,25(OH)_2_D_3_ suggest potential therapeutic avenues for both the endogenous hormone and synthetic vitamin D analogs in the treatment of cancer [3], immune suppression, autoimmune diseases [16,17], and cardiovascular health [18]. In this review, the vitamin D metabolites are defined as the known endogenous bioactive vitamin D compounds, calcitriol and calcidiol, and other synthetic vitamin D analogs.

## 3. Regulation of the Cell Cycle

### 3.1. The Phases of the Cell Cycle

**Figure 1:**
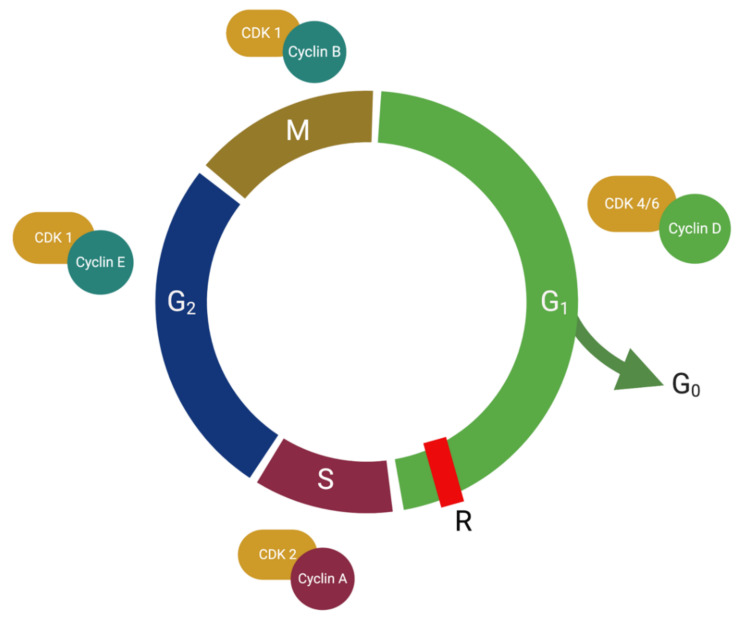
The cell cycle phases and key regulatory checkpoints in eukaryotes. Cells cycle through Gap1 (G1), Gap2 (G2), DNA synthesis (S), and mitosis (M) phases. Cells may temporarily exit the cell cycle at the G1/G0 transition point in response to high cell density or mitogen deprivation. Three cell cycle checkpoints regulate sequential progression between the phases. Regulatory proteins (cyclins) pair with specific catalytic subunits (cyclin-dependent kinases) to form an active kinase that drives cell progression through the restriction point (R). (Abbreviations: G1: Gap1; G2: Gap2; S: Synthesis phase; M; Mitosis; CDK: cyclin-dependent kinase). (Source: personal collection)

All eukaryote organisms grow by undergoing mitotic cell division. The process of cell growth is highly regulated and tightly controlled by the cell cycle. The cell cycle consists of four sequential phases (Figure 1) [19]. Firstly, during the gap1 (G1) phase, the cell is most sensitive to growth signals, and it prepares for cell division by increasing transcription and translation of essential DNA synthesis proteins [19]. Next, the cell enters the S phase where DNA is replicated. Then, the cell progresses to the second gap phase (G2), which allows the cell to prepare the necessary mitotic machinery [19]. Finally, mitosis (M) is initiated, where chromosomes segregate, and the cell divides into two daughter cells. Upon division, the two daughter cells may enter a temporary, quiescent G0 phase, or begin another round of cell cycling, depending on the tissue milieu [20]. The G0 phase of the cell cycle represents a quiescent state in which the cell has transiently withdrawn from the cell cycle and is not actively dividing [19]. The G0 phase is the result of either external stimuli instructing the cell not to divide or decreased mitogen signaling from the tissue environment. Cells in the G0 phase may re-enter the cell cycle by appropriate mitogenic signaling of D-type cyclins [19].

### 3.2. Cyclin-Dependent Kinases and Cyclins Drive the Cell Cycle

Cyclin-dependent kinases (CDKs) are a family of heterodimeric serine/threonine protein kinases that enable the cell to progress sequentially in the cell cycle [20]. CDKs are constitutively expressed catalytic subunits that are activated when bound to their respective cognate cyclin partners [20]. The substrate specificity of the cyclin-CDK complexes is thus dependent on the specific pairing of each cyclin to its cognate CDK [20]. Cyclins are regulatory subunits that are expressed in a cyclical manner throughout the cell cycle [19,20]. Once bound, CDK-cyclin complexes monitor transitions through the phases of the cell cycle. For example, the cyclin D-CDK4 and cyclin D-CDK6 complexes mediate the progression through G1, and the cyclin E-CDK2 complex drives the cell through the restriction point (R point) of G1 [19]. The regulatory checkpoints in the cell cycle are illustrated in Figure 1. Once the cell passes the R point in late-stage G1, it is no longer sensitive to external growth signals and is committed to mitosis [21]. The cell cycles through the S phase by cyclin A-CDK2 complexes. Cyclin B-CDK1 complexes in G2 regulate entry into mitosis [21]. Therefore, selective CDK enzymes are only activated at particular points in the cycle during periods of abundant expression of their cognate regulatory cyclin partner.

### 3.3. The Activation and Inactivation of Cyclin/CDK Complexes Determine the Progression of the Cell Cycle

In mammalian cells, cyclin/CDK complexes require further activation by post-translational modification before they mediate cell cycle progression. Firstly, cyclin activating kinases (CAKs)—a trimolecular complex of Cdk7, cyclin H, and Mat1—phosphorylate Thr160 and secondly, Cdc25 phosphatase dephosphorylates Thr14 and Tyr15 to form an active complex [21]. In contrast, CDKs are inhibited when Thr14 and Tyr15 are phosphorylated by Wee1 and Mik1 kinases, and when bound to cyclin-dependent kinase inhibitors (CDKIs) [21].

CDK inhibitors (CDKIs) finely modulate cell cycling by temporarily blocking the activity of CDK-cyclin complexes [20]. CDKI expression is induced by external stimuli or internal stressors. A wide range of cell cycle inhibitory stimuli exist [20].

The two families of CDKIs are the INK4 and the CYP/Kip inhibitors, and respectively prevent the binding of CDKs to their cognate cyclins, or disrupt activated cyclin-CDK complexes, thereby causing transcriptional inhibition [20,21]. Firstly, INK4 CDKIs are INhibitors of CDK4 and consist of members: p16^INK4A^, p15^INK4B^, p18^INK4C^, and p19^INK4D^. INK4 inhibitors specifically prevent the binding of CDK4 and CDK6 to cyclin D but do not bind other CDKs [20,21]. Secondly, the CIP/KIP family consists of three members: p21^CIP1^ (Cdk Interacting Protein 1), p27^KIP1^ (Kinase Inhibitory Protein 1), and p57^KIP2^ (Kinase Inhibitory Protein 2), which bind CDK2 activity. In this way, CDKIs provide fine regulation of control between the phases of the cell cycle.

### 3.4. Cell Cycle Entry and the G1-S Checkpoint

Mitogenic signals converge on cyclin D-CDK4 and result in the progression of the cell to the restriction point [22]. Cyclin D-CDK4, cyclin D-CDK6, and cyclin E-CDK2 complexes enable progression through the restriction point of the G1 phase of the cell cycle [23]. Once the cell traverses the G1/G0 restriction point, it is committed to cell division and will undergo mitosis [24]. The restriction point is the gatekeeper of the cell cycle [22,25]. For cells to pass the G1/G0 restriction point, compliance with two criteria must be met. Firstly, cyclin E expression requires upregulation, and secondly; the retinoblastoma protein (pRB) must be hyperphosphorylated [26]. These two conditions facilitate the binding of Cdk2 to cyclin E, which then phosphorylates pRB. The hyperphosphorylated pRB then dissociates from E2F transcription factors [26], resulting in free E2F transcription factors that activate cell cycle regulators (cyclins A and E) and DNA synthesis machinery (Mcm, Cdc6, and Cdt1) thus, permitting DNA replication [26].

The pRB pocket protein family includes three members: pRB, p107, and p130, and they are named after a conserved binding pocket region through which pRB, p107, and p130 bind cellular factors such as the E2F family of transcription factors and viral oncoproteins [22]. pRB is a negative regulator of the cell cycle through its repressive interaction with E2F transcription factors. pRB blocks E2F activity in two distinct ways [22]. Firstly, the pRB-E2F complex is sufficient to prohibit the transcriptional activity of E2F, and secondly, the pRb-E2F complex also recruits histone deacetylases (HDACs) to promoter regions of E2F-responsive genes [21,22]. HDACs remove highly charged acetyl moieties from core histones (H3 and H4), and this results in a compacted chromatin architecture between nucleosomes [22] and restricted access of transcription factors to their cognate promoters. The outcome of this pRB-E2F-HDAC repressor complex is a reduction in the transcription of S phase genes [21,22].

#### C-MYC Regulates Numerous Genes in the Cell Cycle

The MYC oncogene family consists of *C-MYC*, *MYCN,* and *MYCL,* which code for the oncoproteins C-MYC, N-MYC, and L-MYC, respectively [27]. These oncoproteins belong to a family of “super transcription factors” which regulate as much as 15% of the human genome [27] involved in cell proliferation, differentiation, metabolism, cell growth, and apoptotic cell death [28,29]. MYC family of proteins consists of highly conserved motifs, and MYC binds to DNA by a basic-region/helix-loop-helix/leucine-zipper (BR/HLH/LZ) motif at the C terminus [28]. The MYC transcription factors recognize and bind to E-box sequences (5′-CACGTG-3′) that are located in the transcriptional regulatory region of target genes, and recruit additional transcription factors [28].

MYC expression is tightly controlled at transcriptional and post-transcriptional levels in healthy cells; however, MYC expression is frequently dysregulated in human cancers [1,27]. C-MYC suppressors the expression of cell cycle inhibitors, such as p15, p21, p27, and GADD-34, -45 and -153 expression [30]. These cell cycle inhibitors prevent unwarranted progression of the G1 phase. Therefore, by decreasing CDKI expression, MYC promotes cell cycle progression and cellular division [31] in pre-malignant and malignant cells and enhances tumor growth and development [32].

However, MYC is considered a double face transcription factor: MYC overexpression promotes cell proliferation in malignancy but also concomitantly sensitizes healthy cells to apoptosis [32]. Healthy cells respond to elevated MYC levels by initiating apoptosis, whereas transformed cells lose their sensitization to MYC levels and often respond to elevated MYC levels by inducing cell proliferation [32,33].

### 3.5. G2/M Checkpoint

Once the cell in the S phase has faithfully replicated its genome, it enters the G2 phase [21]. During this short growth phase, the cell synthesizes requisite proteins for mitosis [21]. The primary objective of the G2 checkpoint is to halt the cell cycle in the presence of DNA damage [21]. Therefore, DNA lesions are not passed to the daughter cells during mitosis. When DNA is damaged, sensor proteins, such as checkpoint kinase 1 (Chk1) and checkpoint kinase 2 (Chk2), initiate the DNA damage response and induce cell cycle arrest [34]. Traverse of G2/M checkpoint is not primarily regulated by CDKIs [35], but instead is controlled by cyclin activating kinase (CAK) [21]. DNA lesions incurred during the S phase are sensed by ATR/Chk1 and ATM/Chk2 pathways and halt the cycle by removing CDK-cyclin activators, Cdc25 phosphatase [21].

## 4. The Effect of Vitamin D Metabolites on the Cell Cycle

Vitamin D derivatives inhibit the progression of the cell cycle in various cell and tumor types. To date, the anti-proliferative action of vitamin D derivatives has been well established in malignant keratinocytes, and this action has also been well studied in breast and prostate cancer cells. The best-described direct action of vitamin D on cell cycle regulators is the role of vitamin D in the G1/G0 cell cycle arrest. The G1/G0 cell cycle arrest is predominantly mediated by the upregulation of CDKIs p21 and p27. The mechanisms of p21 and p27 upregulation by calcitriol are varied. This section will explore the role of vitamin D and its metabolites on p21 and p27 expression. Furthermore, other mechanisms of G1/G0 cell cycle arrest, such as inducing pRB expression and inhibition of C-MYC expression, will also be explored. Lastly, the role of calcitriol in arresting the cell at the G2/M checkpoint will also be discussed.

### 4.1. Upregulation of p21 and p27 by 1,25(OH)2D3

The upregulation of p21 and p27 CDKIs in numerous types of cells treated with vitamin D is frequently documented within in vitro studies (Table 1). Considering the importance of CDKs in driving the cell cycle, it is understandable that an increase in CDKI targeting CDK2 (complexed to cyclin D, E, or A) halts the cell cycle. 

#### 4.1.1. Mechanisms of p21 Upregulation by 1,25(OH)_2_D_3_

**Figure 2:**
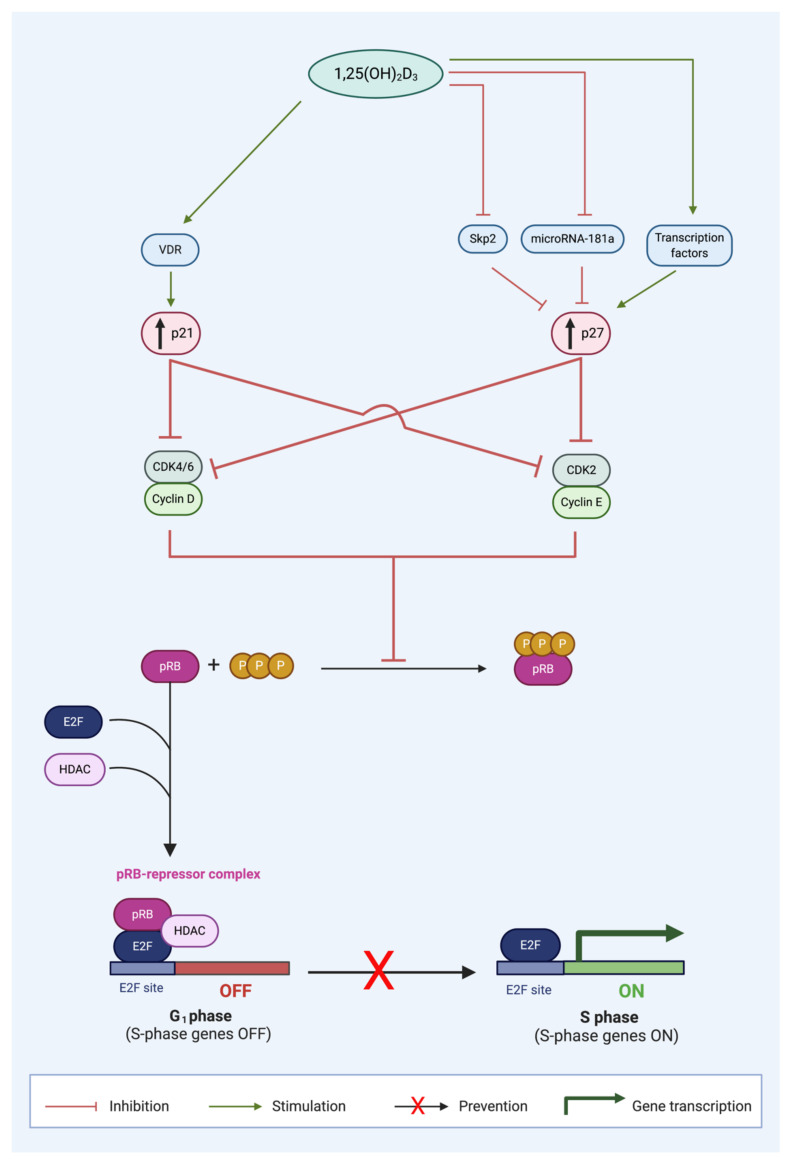
Calcitriol upregulates p21 and p27 expression in the G1 phase and prevents cell cycle progression to the S phase [3]. Calcitriol (1,25(OH)_2_D_3_) increases the expression of CDK inhibitors (CDKIs), p21 and p27, by numerous mechanisms. p21 expression is increased by stimulation of liganded VDR signaling. p27 expression is increased by signaling transcription factors; inhibition of p27 protein degradation by S-phase kinase-associated protein 2 (Skp2); and enhanced p27 translation by abrogated microRNA-181a expression. The collective outcome of the increased CDKI expression is suppression of cyclin-CDK complex formation, which inhibits the formation of hyperphosphorylated retinoblastoma protein (pRB). The unphosphorylated pRB thus is able to form a repressor complex with histone deacetylase (HDAC) and E2F transcription factor (E2F), which prevents the progression of cancer cells in the G1 phase to the S phase, inhibiting S phase gene expression, and thus causing G0/G1 cell cycle arrest. (Source: personal collection).

At a genomic level, 1,25(OH)_2_D_3_ increases the expression of p21 (Figure 2). A functional VDRE has been identified in the *p21* promoter region. This enables direct regulation of *p21* transcription by VDR. p21 is upregulated in cells treated with vitamin D metabolites [56]. Functional VDRE in the p21 promoter has been reported in prostate cancer [46,57], breast cancer [36,38,58], and parathyroid cancer cells [59]. Therefore, VDR binding to VDRE at the *p21* promoter region enhances *p21* transcription and induces cell cycle arrest at G_1_/G_0_.

Within in vitro cell differentiation models, such as myeloid leukemia HL60 and SCC cell lines, p21 showed variable response to calcitriol treatment. In addition to cell cycle arrest, p21 is also associated with cellular differentiation. Despite the presence of VDRE at the p21 gene promoter, studies on calcitriol treatment reveal cell-specific results. In HL60 cells, calcitriol increased p21 expression and induced G1/G0 cell cycle arrest [60]. However, in SCC cells, which are malignant counterparts of keratinocytes, 1,25(OH)_2_D_3_ inhibited cell growth but also decreased p21 expression [61]. In the myelomonocytic cell line, U937, p21 mRNA was significantly increased two hours after treatment with 1,25(OH)_2_D_3,_ and this resulted in a G1 arrest [56]. However, assessment of the cell cycle 24 and 48 h after treatment identified that this initial quick response was not sustained [62]. The initial G1 arrest was followed by a “proliferative burst.” This may suggest that it is unlikely that p21 is solely responsible for the G1/G0 arrest in leukemia. These findings suggest that the role of vitamin D in p21 upregulation and subsequent cell cycle arrest or differentiation remains unclear in healthy and malignant keratinocytes.

In addition, p21 is also transcriptionally regulated by p53 protein [63]. This p53-p21 axis is regulated by multiple p53 binding regions located in the p21 promoter [64]. The p53 tumor-suppressor protein is activated by cellular stressors, for example, oncogene activation [63]. This transcription factor can activate or repress target genes directly by the recruitment of p53 tetramers to response elements on target gene promoter sites to cause tumor suppression by affecting cell cycling and apoptosis [63]. The direct activation of cell cycle arrest proteins by p53 include the upregulation of p21 CDKI [65]. In addition, indirect p53-mediated repression of other tumor-suppressor genes can be affected by the direct p53-dependent increase of p21 expression [66]. Thus, p53 can cause cell cycle disruption directly or indirectly via p21, which recruits E2F4 repression complexes to target promoters of genes involved in cell cycle progression. 1,25(OH)_2_D_3_ has been shown to regulate the p53-p21 axis. Cross-talk between VDR and p53 family members is important in tumor suppression [67]. For example, in gastric cancer cells, multiple sites for p53 binding are present in the promoter region of *p21* and p53 co-operates with VDR to regulate the transactivation of *p21* mRNA [68]. Mechanisms that are important in the cross-talk between vitamin D and p53 signaling include direct regulation of VDR by p53 [69]; the regulation of cutaneous vitamin D synthesis by p53 [70] and the binding of p53 to highly conserved intron sequences of the VDR gene [71]. Additionally, vitamin D metabolites can regulate murine double minute (*MDM2*) gene independent of p53 [72], which encodes an E3 ubiquitin ligase that degrades p53 by the 26S proteosome [73] which can regulate p53-induced cell death. Cell cycle arrest in cancer cell lines has demonstrated p53 dependent and p53 independent mechanisms.

#### 4.1.2. Mechanisms of p27 Upregulation by 1,25(OH)_2_D_3_

In contrast to the genomic regulation of p21, calcitriol regulates p27 at a protein level. In 1996, Wang et al. were the first to report an increase in p27 and subsequent G1 block after 1,25(OH)_2_D_3_ treatment in HL60 cells [43]. In later experiments, the same group demonstrated reduced Cdk6 and Cdk2 kinase activity and p27 upregulation [74] which induced a G1 cell cycle arrest. In addition, p27 silencing by siRNA reversed the G1 block in HL60 cells [75]. Taken together, these findings demonstrate that p27 has a crucial role in G_1_/G_0_ cell cycle arrest in HL60 cells. To date, several different mechanisms of 1,25(OH)_2_D_3_ regulation of p27 have been reported (Figure 2).

The dominant mechanism of p27 regulation by vitamin D is by proteosome-dependent protein degradation. For example, in both ovarian [39] and prostate [76] cancer cell lines, 1,25(OH)_2_D_3_ treatment did not change p27 mRNA levels, but reduced mRNA expression of p45/Skp2 and Cks1 which are responsible for p27 protein ubiquitinylation and degradation [77]. The net effect is that there is decreased ubiquitin tagging of p27 protein and subsequent protein degradation, ultimately leading to increased p27 stabilization [77,78]. This mechanism of p27 stabilization was also observed in acute promyelocytic leukemia cells [79] and human hepatoma cells [80]. By protein stabilization, 1,25(OH)_2_D_3_ may increase p27 expression and sustain the G1 cell cycle block as p27 regulates cyclin E-CDK2 activity [81].

In addition to the aforementioned post-translational mechanism of p27, 1,25(OH)_2_D_3_ also mediates p27 by two transcriptional mechanisms. Firstly, VDR enhanced the expression of transcription factors responsible for the increase in *p27* gene expression, Sp1, and NFY, in SW620 colon cancer cell line and LNCaP prostate cancer cell line [82]. Secondly, calcitriol has been observed to induce Akt expression and thereby indirectly regulate *p27* gene expression [83]. Akt promotes forkhead transcription factors, such as AFX (FOXO4), which is required for p27 gene expression [84,85]. Therefore, VDR indirectly increases p27 expression at the genomic level by several key transcription factors.

Furthermore, 1,25(OH)_2_D_3_ regulates p27 expression by decreasing microRNA 181a expression, and it has been reported in myeloid cells [86]. MicroRNAs repress protein expression at the post-transcriptional level by binding to mRNA transcripts and blocking access to protein synthesis machinery [87]. Wang et al., reported a decrease in expression of miRNA 181a in HL60 and U937 cells when treated with 1,25(OH)_2_D_3_ in a dose- and time-dependent manner [86] which decreased *p27* mRNA whereas cells transfected with pre-miR181a constructs abrogated the 1,25(OH)_2_D_3_-induced upregulation of p27 [86].

Hence, several mechanisms of p27 regulation have been reported, and data suggests a cell-specific response to calcitriol treatment. Additional studies on p27 regulation by vitamin D metabolites in other cell lines and cancer cell types are needed to fully elucidate the mechanism of p27 upregulation by vitamin D metabolites in cancer.

### 4.2. The Effect of 1,25(OH)_2_D_3_ on pRB Expression

By increasing CDKIs p21 and p27, 1,25(OH)_2_D_3_ directly decreases the action of downstream targets, cyclin D-CDK4/6, and cyclin E-CDK2 complexes, respectively. The result of these perturbations of cell cycle regulatory proteins is that pRB remains in a hypophosphorylated state complexed to E2F [21]. In this way, 1,25(OH)_2_D_3_ prevents the transcriptional activity of E2F transcription factors and denies entry into the S phase of the cell cycle. In addition to this indirect role of 1,25(OH)_2_D_3_ on E2F expression, 1,25(OH)_2_D_3_ can increase the expression of pRB in myeloid leukemia cells approximately 10 h after vitamin D treatment [88], although the mechanism of pRB upregulation is currently unknown. The increased abundance of pRB sequesters any free E2F factors and inhibits E2F, which results in a G1/G0 cell cycle arrest [21].

### 4.3. Downregulation of C-MYC by 1,25(OH)_2_D_3_

In addition to its regulation of pRB, 1,25(OH)2D3 has demonstrated downregulation of the prominent pRB target gene, C-MYC. Several studies have demonstrated a significant reduction of C-MYC expression after 1,25(OH)2D3 treatment in colon [89,90] and prostate cancer cell lines [48,91]. These studies collectively demonstrate that decreased C-MYC expression is associated with G1/G0 arrest, followed by cell differentiation.

The mechanism for C-MYC repression by calcitriol seems to be cell-specific. In prostate cancer C4-2 cells, 1,25(OH)_2_D_3_ treatment reduced C-MYC mRNA by 50% and resulted in a significant reduction in C-MYC protein [48]. In SW480 colon cancer cells, 1,25(OH)_2_D_3_ promoted VDR/β-catenin interaction and prevented the β-catenin translocation into the nucleus [92]. β-catenin is important for C-MYC gene transcription. Thus, 1,25(OH)_2_D_3_ may indirectly inhibit *C-MYC* gene transcription via the APC/β-catenin pathway. C-MYC therefore appears to be an important target of calcitriol in policing cancer cells to cause G1/G0 arrest.

### 4.4. Role of 1,25(OH)_2_D_3_ in G2/M Cell Cycle Arrest

Currently, there is limited evidence of the induction of G2/M arrest by 1,25(OH)_2_D_3_, with the general consensus that G1/G0 cell cycle arrest is the primary target of vitamin D metabolites. However, there are a selected number of studies that have shown G2/M cell cycle arrest in cells treated with 1,25(OH)_2_D_3_. In HL60 myeloid leukemia cells treated with 1,25(OH)_2_D_3_, a G2/M arrest was evident in cell cycle analyses [93,94]. Only one study identified a mechanistic link in 1,25(OH)_2_D_3_-induced G2/M cell cycle arrest. In ovarian cancer cell lines, 1,25(OH)_2_D_3_ increased GADD45α (Growth arrest and DNA-Damage-Inducible alpha) and subsequently induced cell cycle arrest at the G2/M phase [95]. Interestingly, a functional VDRE was identified in an exonic enhancer region of the *GADD45α* gene [95]; however, no additional studies exploring GADD45α, and this VDRE in other cancer cell lines, have been identified.

### 4.5. Epigenetic Marks of the Vitamin D Metabolizing System in Cancer Cell Lines May Alter Cell Arrest

The VDMS, at an autocrine level, controls genes that regulate cell proliferation and cell death. CpG islands span the promoters of CYP2R1, CYP24A1, and VDR, while a CpG island is located within the CYP27B1 gene locus [96].

Liganded VDR signaling has been shown to be attenuated in cancer [96]. Epigenetic silencing of the VDR can be mediated by hypermethylation in various types of cancerous cells, including breast and choriocarcinoma tumor cell lines [97]. The pattern of hypermethylation of the VDR promoter region is inconsistent in cancer cell lines; for example, colonic cancer cells do not reveal a hypermethylated status in the VDR promoter region [98,99]. Therefore, the decreased sensitivity to calcitriol may be caused by epigenetic corruption of the VDR in cancer cell lines. In addition, epigenetic marks can reduce sensitivity to vitamin D metabolites in clinical trials [100] by increased methylation of VDR promoter and associated VDR expression in cancer [101].

In addition, expression of CYP27B1 is often downregulated in cancer which may be accounted for by CpG islands also present in the gene locus. In breast cancer cell lines, the CYP27B1 gene showed reversible DNA hypermethylation which caused CYP27B1 silencing. Similarly, Novakovic et al. [102] demonstrated hypermethylation of the CYP27B1 gene promoter region in the choriocarcinoma cell lines, BeWo and JAR. Inhibitors of methylation in prostate cancer cell lines increased CYP27B1 expression [103] supporting the importance of epigenetic marking of CYP27B1 in autocrine calcitriol synthesis. Hypermethylation of CYP27B1, therefore, may be associated with the decreased local synthesis of calcitriol from calcidiol substrate, and potentially decreased cell growth, differentiation, and perturbed cell cycling.

The regulation of CYP24A1 by DNA methylation demonstrates cell-specificity. Hypermethylation of the CYP24A1 promoter region, with associated epigenetic silencing of CYP24A1 expression, has been identified in prostate cancer cell line PC3 [104] and tumor-derived endothelial cells (TDEC) [105]. The hypomethylation of the CYP24A1 promoter in colon adenocarcinoma associated with significantly elevated CYP24A1 expression [106] has also been observed. The catabolic role of CYP24A1 in these studies may support the disruption of calcitriol-mediated cell arrest in tumorigenic cell lines.

Collectively, these studies demonstrate that the epigenetic regulation of gene expression of the VDMS is altered in cancer mainly by DNA hypermethylation. This altered state leads to abnormal protein expression levels, which may favor carcinogenesis in a cell-specific manner. Therefore, autocrine regulation of the VDMS may impact G1/G0 and G2/M cell cycle arrest in cell lines and in clinical studies. Epigenetic alteration of the VDMS may also provide insight into the discordant alignment between in vitro cancer studies and clinical studies. Exploration of epigenetic marks of the VDMS on cell cycle regulators may thus provide a possible explanation of the inhibition of cell arrest in cancer patients. Future studies can also target altered autocrine activation of vitamin D precursors, autocrine catabolism of activated vitamin D hormone, and abrogated signaling of liganded VDR, and their collective association with perturbations of vitamin D-induced cell cycle arrest.

## 5. Conclusions

Vitamin D metabolites target numerous cell cycle regulators in order to arrest the cell cycle. G1/G0 arrest is the primary mode of cell cycle arrest described within in vitro cell lines treated with vitamin D compounds, although a few studies have described G2/M cell cycle block with vitamin D treatments. Within the G1/G0 arrest, several studies have demonstrated the upregulation of CDKIs, p21, and p27, while few studies have also shown decreased C-MYC expression and increased pRB expression. In addition, the mechanisms of cell cycle arrest by vitamin D demonstrate cell specificity. More data are needed to fully elucidate the mechanistic action of vitamin D on cell cycle proteins, especially to resolve the role of vitamin D metabolites’ anti-cancer actions in pre-clinical and clinical studies. The altered vitamin D metabolizing system by epigenetic marks may partly account for dysregulated vitamin D activation and signaling, and studies exploring this topic may provide insight into the perturbations of cell cycle arrest in cancer. The exploitation of cell arrest mechanisms by vitamin D and its analogs is a promising though currently unresolved therapeutic target. 

## Figures and Tables

**Table 1:** Mechanisms of cell cycle arrest induced by calcitriol and/or vitamin D analogues on cancer cell lines.

Tissue of Origin	Author	Cell Line/s	Treatment (Concentration)	Mechanism of Action	Conclusion
Breast cancer	S. Jensen et al. [36]	MCF-7	1,25(OH)_2_D_3_ (100 nM)	1,25(OH)_2_D_3_ increased tumor suppressor pRB expression and decreased expression of CDK 4, 6 and 2 and increased expression of CDKI p21. 1,25(OH)_2_D_3_ treatment also decreased C-MYC oncoprotein expression.	G0/G1 cell cycle arrest
	Chiang et al. [37]	MCF-7	1. MART-10 (1 nM, 10 nM and 100 nM) 2. 1,25(OH)_2_D_3_ (10 nM, 100 nM and 1000 nM)	1,25(OH)_2_D_3_ and MART-10 induced p21 and p27 CDKI expression and induced G0/G1 cell cycle arrest.	G0/G1 cell cycle arrest
	Wu et al. [38]	MCF-7 E MCF-7 L BT20 T47D ZR75	EB1089 (0.01 nM, 0.1 nM, 1 nM, 10 nM)	EB1089 induced p21 expression and increased p21-CDK2 complex formation, which caused decreased DNA synthesis in all cell lines except EB1089-resistant MCF-7 L cell line. p27 was increased by EB1089 treatment in BT20 and ZR75 cell lines only.	Cell-dependent G0/G1 cell cycle arrest
Ovarian cancer	Li et al. [39]	2008 CAOV3	1,25(OH)_2_D_3_ (100 nM)	1,25(OH)_2_D_3_ decreased the expression of cyclin E and Skp2, which resulted in decreased CDK2-cyclin E activity and decreased p27 phosphorylation, respectively. The decreased p27 phosphorylation prevents p27 protein degradation, allowing it to accumulate in the cell and induce G1/G0 cell cycle arrest.	G0/G1 cell cycle arrest
	Li et al. [39]	OVCAR3	1,25(OH)_2_D_3_ (100 nM)	VDR stabilized intracellular p27 protein levels by decreasing the activity of the Skp2 proteosome, which is responsible for p27 degradation.	G0/G1 cell cycle arrest
Human head and neck squamous cells	Akutsu et al. [40]	SCC25	EB1089 (1 nM, 10 nM and 100 nM)	Calcitriol analogue EB1089 upregulated growth repair damage factor GADD45α.	G0/G1 cell cycle arrest
	Salehi-Tabar et al. [41]	SCC25	1,25(OH)_2_D_3_ (100 nM)	1,25(OH)_2_D_3_ decreased C-MYC expression and increased C-MYC repressor MAD1 levels. The increased MAD1 prevented C-MYC’s transcriptional regulation of target genes and inhibited cell proliferation.	G0/G1 cell cycle arrest
Thyroid cancer	Liu et al. [42]	PTC-1 NPA WRO	1. 1,25(OH)_2_D_3_ (0.1 nM, 1 nM, 10 nM, 100 nM and 1000 nM) 2. EB1089 (0.1 nM, 1 nM, 10 nM, 100 nM and 1000 nM)	1,25(OH)_2_D_3_ and EB1089 increased p27 expression and decreased Skp2 expression, which allowed p27 to accumulate and induce G0/G1 cell cycle arrest.	G0/G1 cell cycle arrest
Promyelocytic leukaemia	Wang et al. [43]	HL60	1,25(OH)_2_D_3_ (1 nM and 100 nM)	1,25(OH)_2_D_3_ induced p12 and p27 mRNA and protein expression and induced G0/G1 cell cycle arrest.	G0/G1 cell cycle arrest
Prostate cancer	Washington et al. [44]	C4-2	1,25(OH)_2_D_3_ (100 nM)	1,25(OH)_2_D_3_ decreased C-MYC expression and induced G1 cell cycle arrest in a pRB-independent manner.	G0/G1 cell cycle arrest
	Bao et al. [45]	LNCaP CWR22R PC-3 DU145	1,25(OH)_2_D_3_ (100 nM)	1,25(OH)_2_D_3_ increased pRB and p27 expression and decreased CDK2 expression, thereby preventing entry into the S phase.	G0/G1 cell cycle arrest
	Boyle et al. [46]	LNCaP	1,25(OH)_2_D_3_ (10 nM)	Calcitriol upregulated the mRNA and protein expression of insulin-like growth factor binding protein 3, which resulted in increased expression of p21 and induced a G0/G1 cell cycle arrest.	G0/G1 cell cycle arrest
	Flores et al. [47]	LNCaP	1,25(OH)_2_D_3_ (50 nM)	1,25(OH)_2_D_3_ decreased CDK2 activity leading to hypophosphorylation of pRB, which prevented entry into the S phase.	G0/G1 cell cycle arrest
	Rohan et al. [48]	LnCaP C4-2 RWPE-1	1,25(OH)_2_D_3_ (10 nM)	Downregulation of C-MYC mRNA and protein expression induced by 1,25(OH)_2_D_3_ treatment.	G0/G1 cell cycle arrest
Colorectal adenoma and carcinoma	Diaz et al. [49]	SW620 PC/JW HT29	1. 1,25(OH)_2_D_3_ (0.1 nM, 1 nM, 10 nM, 100 nM, 1000 nM) 2. EB1089 (0.1 nM, 1 nM, 10 nM, 100 nM, 1000 nM)	Calcitriol and analogue EB1089 increased cells in G1 in a p53- dependent manner.	G0/G1 cell cycle arrest
Pancreatic cancer	Li et al. [50]	HPDE6-C7 Panc-1	1,25(OH)_2_D_3_ (1 nM, 5 nM, 10 nM, 50 nM, 100 nM)	p21 expression was significantly increased in HPDE6-C7 cells but not in metastatic Panc-1 cells.	G0/G1 cell cycle arrest
	Petterson et al. [51]	AsPc-1 BxPc-3 T3M-4	1. EB1089 (50 nM) 2. CB1093 (50 nM)	EB1089 and CB1093 induced cell cycle arrest in all cell lines investigated in this study.	G_0_/G_1_ cell cycle arrest
	Schwartz et al. [52]	BxPC-3 Hs700T Hs766T AsPC-1	1. 1,25(OH)_2_D_3_ (100 nM) 2. 25(OH)D_3_ (100 nM or 2µM)	Increased expression of p21 and p27 proteins in BxPC-3, Hs700T and AsPC-1 cell lines only.	G0/G1 cell cycle arrest
Malignant pleural mesothelioma	Gesmundo et al. [53]	MeT-5A Msto-211H REN	1,25(OH)_2_D_3_ (1 nM, 10 nM, 50 nM and 100 nM)	Reduction in C-MYC expression and *cyclin A*, *cyclin D1* and *cyclin D2* which induced a G1/G0 cell cycle arrest.	G0/G1 cell cycle arrest
Malignant melanoma	Reichrath et al. [54]	IGR MelJuso MeWo SK-Mel-5 SK-Mel-25 SK-Mel-28 SM2	1. 1,25(OH)_2_D_3_ (100 nM) 2. 25(OH)D_3_ (100 nM) 3. EB1089 (100 nM)	Treatments induced a significant decrease in cell proliferation of MeWo, SK-Mel 28, and SM2 melanoma cell lines. In addition, IGR, MelJuso, SkMel5 and SK-Mel-25 cell lines demonstrated no significant change in cell growth.	Cell cycle not investigated; however, a significant decrease in cell proliferation was observed in a cell-specific manner.
	Spath et al. [55]	IR6 VAG 1007	1,25(OH)_2_D_3_ (50 nM)	1,25(OH)_2_D_3_ induced G1/G0 cell cycle arrest in IR6 cell line by p21 and p27 upregulation, and cyclin D downregulation. 1,25(OH)_2_D_3_ induced G2/M arrest in VAG cell line by decreased cyclin B1 expression. In 1007 melanoma cell line, 1,25(OH)_2_D_3_ increased cells in the proliferative compartments of the cell cycle (S-phase plus G2 phase) by increased cyclin A1, p21 and p27 expression.	Cell-specific cell arrest responses were observed to 1,25(OH)_2_D_3_ treatment.
	Liu et al. [56]	U937	1,25(OH)_2_D_3_ (100 nM)	1,25(OH)_2_D_3_ induced p21 mRNA expression in a p53-independent manner and 1,25(OH)_2_D_3_ induced p27 gene and protein expression.	1,25(OH)_2_D_3_ arrested cell proliferation and induced cell surface markers of cell differentiation.

Abbreviations: 1,25(OH)_2_D_3_, calcitriol; 25(OH)_2_D_3_, calcidiol; EB1089, Seocalcitol; CB1093, novel 20-epi-vitamin D_3_ analogue; MART-10 (19-nor-2α-(3-hydroxypropyl)-1α,25(OH)₂D₃; pRB, retinoblastoma protein.

## Abbreviations

CDK	Cyclin-dependent kinase
CDKI	Cyclin-dependent kinase inhibitor
CIP	Cdk Interacting Protein
Chk	Checkpoint kinase
G_0_ phase	Resting (quiescent) phase
G_1_ phase	Gap 1 phase
G_2_ phase	Gap 2 phase
GADD	Growth arrest and DNA-damage-inducible protein
HDAC	Histone deacetylase
KIP	Kinase Inhibitory Protein
M phase	Mitosis
MDM2	Murine Double Minute 2
pRB	Retinoblastoma protein
S phase	Synthesis
Skp2	S-phase kinase-associated protein 2
TDEC	Tumor-derived endothelial cells
VDR	Vitamin D receptor
VDRE	Vitamin D response element

## References

[1] Hanahan D., Weinberg R.A. (2011). Hallmarks of Cancer: The Next Generation. Cell.

[2] Bray F., Ferlay J., Soerjomataram I., Siegel R.L., Torre L.A., Jemal A. (2018). Global cancer statistics 2018: GLOBOCAN estimates of incidence and mortality worldwide for 36 cancers in 185 countries. CA Cancer J. Clin..

[3] Feldman D., Krishnan A.V., Swami S., Giovannucci E., Feldman B.J. (2014). The role of vitamin D in reducing cancer risk and progression. Nat. Rev. Cancer.

[4] Fleet J.C., DeSmet M., Johnson R., Li Y. (2012). Vitamin D and cancer: A review of molecular mechanisms. Biochem. J..

[5] Garland C.F., Garland F.C. (1980). Do sunlight and vitamin D reduce the likelihood of colon cancer?. Int. J. Epidemiol..

[6] Colston K., Colston M.J., Feldman D. (1981). 1,25-dihydroxyvitamin D3 and malignant melanoma: The presence of receptors and inhibition of cell growth in culture. Endocrinology.

[7] Giammanco M., Di Majo D., La Guardia M., Aiello S., Crescimannno M., Flandina C., Tumminello F.M., Leto G. (2015). Vitamin D in cancer chemoprevention. Pharm. Biol..

[8] Bhoora S., Pather Y., Marais S., Punchoo R. (2020). Cholecalciferol Inhibits Cell Growth and Induces Apoptosis in the CaSki Cell Line. Med. Sci..

[9] Trump D., Aragon-Ching J. (2018). Vitamin D in prostate cancer. Asian J. Androl..

[10] Campbell M.J., Trump D.L. (2017). Vitamin D Receptor Signaling and Cancer. Endocrinol. Metab. Clin. N. Am..

[11] Holick M.F. (2007). Vitamin D Deficiency. N. Engl. J. Med..

[12] Jenkinson C. (2019). The vitamin D metabolome: An update on analysis and function. Cell Biochem. Funct..

[13] Ozono K., Liao J., Kerner S.A., Scott R.A., Pike J.W. (1990). The vitamin D-responsive element in the human osteocalcin gene. Association with a nuclear proto-oncogene enhancer. J. Biol. Chem..

[14] Bouillon R., Carmeliet G., Verlinden L., van Etten E., Verstuyf A., Luderer H.F., Lieben L., Mathieu C., Demay M. (2008). Vitamin D and Human Health: Lessons from Vitamin D Receptor Null Mice. Endocr. Rev..

[15] Deeb K.K., Trump D.L., Johnson C.S. (2007). Vitamin D signalling pathways in cancer: Potential for anti-cancer therapeutics. Nat. Rev. Cancer.

[16] Cantorna M.T., Zhu Y., Froicu M., Wittke A. (2004). Vitamin D status, 1,25-dihydroxyvitamin D3, and the immune system. Am. J. Clin. Nutr..

[17] Ponsonby A.L., McMichael A., van der Mei I. (2002). Ultraviolet radiation and autoimmune disease: Insights from epidemiological research. Toxicology.

[18] Zittermann A. (2006). Vitamin D and disease prevention with special reference to cardiovascular disease. Prog. Biophys. Mol. Biol..

[19] Williams G.H., Stoeber K. (2012). The cell cycle and cancer. J. Pathol..

[20] Sánchez I., Dynlacht B.D. (2005). New insights into cyclins, CDKs, and cell cycle control. Semin. Cell Dev. Biol..

[21] Malumbres M., Niederhuber J.E., Armitage J.O., Kastan M.B., Doroshow J.H., Tepper J.E. (2020). 4-Control of the Cell Cycle. Abeloff’s Clinical Oncology.

[22] Cobrinik D. (2005). Pocket proteins and cell cycle control. Oncogene.

[23] Malumbres M., Barbacid M. (2005). Mammalian cyclin-dependent kinases. Trends Biochem. Sci..

[24] Sherr C.J., Roberts J.M. (2004). Living with or without cyclins and cyclin-dependent kinases. Genes Dev..

[25] Kastan M.B., Bartek J. (2004). Cell-cycle checkpoints and cancer. Nature.

[26] Nevins J.R. (2001). The Rb/E2F pathway and cancer. Hum. Mol. Genet..

[27] Bretones G., Delgado M.D., León J. (2015). Myc and cell cycle control. Biochim. Biophys. Acta (Bba) Gene Regul. Mech..

[28] Beaulieu M.-E., Castillo F., Soucek L. (2020). Structural and Biophysical Insights into the Function of the Intrinsically Disordered Myc Oncoprotein. Cells.

[29] Dang C.V., O’Donnell K.A., Zeller K.I., Nguyen T., Osthus R.C., Li F. (2006). The c-Myc target gene network. Seminars in Cancer Biology.

[30] Gartel A.L., Shchors K. (2003). Mechanisms of c-myc-mediated transcriptional repression of growth arrest genes. Exp. Cell Res..

[31] Yap C.-S., Peterson A.L., Castellani G., Sedivy J.M., Neretti N. (2011). Kinetic profiling of the c-Myc transcriptome and bioinformatic analysis of repressed gene promoters. Cell Cycle.

[32] Vecchio E., Fiume G., Correnti S., Romano S., Iaccino E., Mimmi S., Maisano D., Nisticò N., Quinto I. (2020). Insights about MYC and Apoptosis in B-Lymphomagenesis: An Update from Murine Models. Int. J. Mol. Sci..

[33] Meyer N., Kim S.S., Penn L.Z. (2006). The Oscar-worthy role of Myc in apoptosis. Semin. Cancer Biol..

[34] Smith J., Tho L.M., Xu N., Gillespie D.A. (2010). The ATM-Chk2 and ATR-Chk1 pathways in DNA damage signaling and cancer. Adv. Cancer Res..

[35] DiPaola R.S. (2002). To arrest or not to G(2)-M Cell-cycle arrest: Commentary re: A. K. Tyagi et al., Silibinin strongly synergizes human prostate carcinoma DU145 cells to doxorubicin-induced growth inhibition, G(2)-M arrest, and apoptosis. Clin. Cancer Res. 8: 3512–3519, 2002. Clin. Cancer Res..

[36] Jensen S.S., Madsen M.W., Lukas J., Binderup L., Bartek J. (2001). Inhibitory effects of 1alpha,25-dihydroxyvitamin D(3) on the G(1)-S phase-controlling machinery. Mol. Endocrinol..

[37] Chiang K.-C., Yeh C.-N., Chen S.-C., Shen S.-C., Hsu J.-T., Yeh T.-S., Pang J.-H.S., Su L.-J., Takano M., Kittaka A. (2012). MART-10, a New Generation of Vitamin D Analog, Is More Potent than 1α,25-Dihydroxyvitamin D3 in Inhibiting Cell Proliferation and Inducing Apoptosis in ER+ MCF-7 Breast Cancer Cells. Evid. Based Complement. Altern. Med..

[38] Wu G., Fan R.S., Li W., Ko T.C., Brattain M.G. (1997). Modulation of cell cycle control by vitamin D3 and its analogue, EB1089, in human breast cancer cells. Oncogene.

[39] Li P., Li C., Zhao X., Zhang X., Nicosia S.V., Bai W. (2004). p27(Kip1) stabilization and G(1) arrest by 1,25-dihydroxyvitamin D(3) in ovarian cancer cells mediated through down-regulation of cyclin E/cyclin-dependent kinase 2 and Skp1-Cullin-F-box protein/Skp2 ubiquitin ligase. J. Biol. Chem..

[40] Akutsu N., Lin R., Bastien Y., Bestawros A., Enepekides D.J., Black M.J., White J.H. (2001). Regulation of gene Expression by 1alpha,25-dihydroxyvitamin D3 and Its analog EB1089 under growth-inhibitory conditions in squamous carcinoma Cells. Mol. Endocrinol..

[41] Salehi-Tabar R., Nguyen-Yamamoto L., Tavera-Mendoza L.E., Quail T., Dimitrov V., An B.-S., Glass L., Goltzman D., White J.H. (2012). Vitamin D receptor as a master regulator of the c-MYC/MXD1 network. Proc. Natl. Acad. Sci. USA.

[42] Liu W., Asa S.L., Fantus I.G., Walfish P.G., Ezzat S. (2002). Vitamin D arrests thyroid carcinoma cell growth and induces p27 dephosphorylation and accumulation through PTEN/akt-dependent and -independent pathways. Am. J. Pathol..

[43] Wang Q.M., Jones J.B., Studzinski G.P. (1996). Cyclin-dependent kinase inhibitor p27 as a mediator of the G1-S phase block induced by 1,25-dihydroxyvitamin D3 in HL60 cells. Cancer Res..

[44] Washington M.N., Kim J.-S., Weigel N.L. (2011). 1α,25-dihydroxyvitamin D3 inhibits C4-2 prostate cancer cell growth via a retinoblastoma protein (Rb)-independent G1 arrest. Prostate.

[45] Bao B.Y., Hu Y.C., Ting H.J., Lee Y.F. (2004). Androgen signaling is required for the vitamin D-mediated growth inhibition in human prostate cancer cells. Oncogene.

[46] Boyle B.J., Zhao X.Y., Cohen P., Feldman D. (2001). Insulin-like growth factor binding protein-3 mediates 1 alpha,25-dihydroxyvitamin d(3) growth inhibition in the LNCaP prostate cancer cell line through p21/WAF1. J. Urol..

[47] Flores O., Wang Z., Knudsen K.E., Burnstein K.L. (2010). Nuclear targeting of cyclin-dependent kinase 2 reveals essential roles of cyclin-dependent kinase 2 localization and cyclin E in vitamin D-mediated growth inhibition. Endocrinology.

[48] Rohan J.N., Weigel N.L. (2009). 1α,25-dihydroxyvitamin D3 reduces c-Myc expression, inhibiting proliferation and causing G1 accumulation in C4-2 prostate cancer cells. Endocrinology.

[49] Diaz G.D., Paraskeva C., Thomas M.G., Binderup L., Hague A. (2000). Apoptosis is induced by the active metabolite of vitamin D3 and its analogue EB1089 in colorectal adenoma and carcinoma cells: Possible implications for prevention and therapy. Cancer Res..

[50] Li L., Shang F., Zhu Y., Sun Y., Sudi R.S. (2019). Modulation of VDR and Cell Cycle-Related Proteins by Vitamin D in Normal Pancreatic Cells and Poorly Differentiated Metastatic Pancreatic Cancer Cells. Nutr. Cancer.

[51] Pettersson F., Colston K.W., Dalgleish A.G. (2000). Differential and antagonistic effects of 9-cis-retinoic acid and vitamin D analogues on pancreatic cancer cells in vitro. Br. J. Cancer.

[52] Schwartz G.G., Eads D., Rao A., Cramer S.D., Willingham M.C., Chen T.C., Jamieson D.P., Wang L., Burnstein K.L., Holick M.F. (2004). Pancreatic cancer cells express 25-hydroxyvitamin D-1 alpha-hydroxylase and their proliferation is inhibited by the prohormone 25-hydroxyvitamin D3. Carcinogenesis.

[53] Gesmundo I., Silvagno F., Banfi D., Monica V., Fanciulli A., Gamba G., Congiusta N., Libener R., Riganti C., Ghigo E. (2020). Calcitriol Inhibits Viability and Proliferation in Human Malignant Pleural Mesothelioma Cells. Front. Endocrinol..

[54] Reichrath J., Rech M., Moeini M., Meese E., Tilgen W., Seifert M. (2007). In vitro comparison of the vitamin D endocrine system in 1,25(OH)2D3-responsive and -resistant melanoma cells. Cancer Biol. Ther..

[55] Spath L., Ulivieri A., Lavra L., Fidanza L., Carlesimo M., Giubettini M., Narcisi A., Luciani E., Bucci B., Pisani D. (2017). Antiproliferative Effects of 1α-OH-vitD3 in Malignant Melanoma: Potential Therapeutic implications. Sci. Rep..

[56] Liu M., Lee M.H., Cohen M., Bommakanti M., Freedman L.P. (1996). Transcriptional activation of the Cdk inhibitor p21 by vitamin D3 leads to the induced differentiation of the myelomonocytic cell line U937. Genes Dev..

[57] Moffatt K.A., Johannes W., Hedlund T., Miller G. (2001). Growth inhibitory effects of 1alpha, 25-dihydroxyvitamin D(3) are mediated by increased levels of p21 in the prostatic carcinoma cell line ALVA-31. Cancer Res..

[58] Verlinden L., Verstuyf A., Convents R., Marcelis S., Van Camp M., Bouillon R. (1998). Action of 1,25(OH)2D3 on the cell cycle genes, cyclin D1, p21 and p27 in MCF-7 cells. Mol. Cell. Endocrinol..

[59] Cozzolino M., Lu Y., Finch J., Slatopolsky E., Dusso A.S. (2001). p21WAF1 and TGF-alpha mediate parathyroid growth arrest by vitamin D and high calcium. Kidney Int..

[60] Hager G., Kornfehl J., Knerer B., Weigel G., Formanek M. (2004). Molecular analysis of p21 promoter activity isolated from squamous carcinoma cell lines of the head and neck under the influence of 1,25(OH)2 vitamin D3 and its analogs. Acta Otolaryngol..

[61] Hershberger P.A., Modzelewski R.A., Shurin Z.R., Rueger R.M., Trump D.L., Johnson C.S. (1999). 1,25-Dihydroxycholecalciferol (1,25-D3) inhibits the growth of squamous cell carcinoma and down-modulates p21(Waf1/Cip1) in vitro and in vivo. Cancer Res..

[62] Rots N.Y., Iavarone A., Bromleigh V., Freedman L.P. (1999). Induced differentiation of U937 cells by 1,25-dihydroxyvitamin D3 involves cell cycle arrest in G1 that is preceded by a transient proliferative burst and an increase in cyclin expression. Blood.

[63] Vousden K.H., Prives C. (2009). Blinded by the Light: The Growing Complexity of p53. Cell.

[64] Saramäki A., Banwell C.M., Campbell M.J., Carlberg C. (2006). Regulation of the human p21(waf1/cip1) gene promoter via multiple binding sites for p53 and the vitamin D3 receptor. Nucleic Acids Res..

[65] Laptenko O., Prives C. (2006). Transcriptional regulation by p53: One protein, many possibilities. Cell Death Differ..

[66] Benson E.K., Mungamuri S.K., Attie O., Kracikova M., Sachidanandam R., Manfredi J.J., Aaronson S.A. (2014). p53-dependent gene repression through p21 is mediated by recruitment of E2F4 repression complexes. Oncogene.

[67] Reichrath J., Reichrath S., Heyne K., Vogt T., Roemer K. (2014). Tumor suppression in skin and other tissues via cross-talk between vitamin D- and p53-signaling. Front. Physiol..

[68] Li M., Li L., Zhang L., Hu W., Shen J., Xiao Z., Wu X., Chan F.L., Cho C.H. (2017). 1,25-Dihydroxyvitamin D(3) suppresses gastric cancer cell growth through VDR- and mutant p53-mediated induction of p21. Life Sci..

[69] Maruyama R., Aoki F., Toyota M., Sasaki Y., Akashi H., Mita H., Suzuki H., Akino K., Ohe-Toyota M., Maruyama Y. (2006). Comparative genome analysis identifies the vitamin D receptor gene as a direct target of p53-mediated transcriptional activation. Cancer Res..

[70] Yamaguchi Y., Hearing V.J. (2009). Physiological factors that regulate skin pigmentation. Biofactors.

[71] Kommagani R., Payal V., Kadakia M.P. (2007). Differential regulation of vitamin D receptor (VDR) by the p53 Family: p73-dependent induction of VDR upon DNA damage. J. Biol. Chem..

[72] Chen J., Marechal V., Levine A.J. (1993). Mapping of the p53 and mdm-2 interaction domains. Mol. Cell. Biol..

[73] Roemer K. (2012). Notch and the p53 clan of transcription factors. Adv. Exp. Med. Biol..

[74] Wang Q.M., Luo X., Studzinski G.P. (1997). Cyclin-dependent kinase 6 is the principal target of p27/Kip1 regulation of the G1-phase traverse in 1,25-dihydroxyvitamin D3-treated HL60 cells. Cancer Res..

[75] Wang Q.M., Chen F., Luo X., Moore D.C., Flanagan M., Studzinski G.P. (1998). Lowering of p27Kip1 levels by its antisense or by development of resistance to 1,25-dihydroxyvitamin D3 reverses the G1 block but not differentiation of HL60 cells. Leukemia.

[76] Yang E.S., Burnstein K.L. (2003). Vitamin D Inhibits G1 to S Progression in LNCaP Prostate Cancer Cells through p27Kip1 Stabilization and Cdk2 Mislocalization to the Cytoplasm. J. Biol. Chem..

[77] Tsvetkov L.M., Yeh K.H., Lee S.J., Sun H., Zhang H. (1999). p27(Kip1) ubiquitination and degradation is regulated by the SCF(Skp2) complex through phosphorylated Thr187 in p27. Curr. Biol..

[78] Carrano A.C., Eytan E., Hershko A., Pagano M. (1999). SKP2 is required for ubiquitin-mediated degradation of the CDK inhibitor p27. Nat. Cell Biol..

[79] Lin R., Wang T.T., Miller W.H., White J.H. (2003). Inhibition of F-Box protein p45(SKP2) expression and stabilization of cyclin-dependent kinase inhibitor p27(KIP1) in vitamin D analog-treated cancer cells. Endocrinology.

[80] Luo W., Chen Y., Liu M., Du K., Zheng G., Cai T., Zhang W., Zhao F., Yao T., Yang R. (2009). EB1089 Induces Skp2-Dependent p27 Accumulation, Leading to Cell Growth Inhibition and Cell Cycle G1 Phase Arrest in Human Hepatoma Cells. Cancer Investig..

[81] Moller M.B. (2000). P27 in cell cycle control and cancer. Leuk. Lymphoma.

[82] Huang Y.C., Chen J.Y., Hung W.C. (2004). Vitamin D3 receptor/Sp1 complex is required for the induction of p27Kip1 expression by vitamin D3. Oncogene.

[83] Zhang Y., Zhang J., Studzinski G.P. (2006). AKT pathway is activated by 1, 25-dihydroxyvitamin D3 and participates in its anti-apoptotic effect and cell cycle control in differentiating HL60 cells. Cell Cycle.

[84] Medema R.H., Kops G.J., Bos J.L., Burgering B.M. (2000). AFX-like Forkhead transcription factors mediate cell-cycle regulation by Ras and PKB through p27kip1. Nature.

[85] Stahl M., Dijkers P.F., Kops G.J., Lens S.M., Coffer P.J., Burgering B.M., Medema R.H. (2002). The forkhead transcription factor FoxO regulates transcription of p27Kip1 and Bim in response to IL-2. J. Immunol..

[86] Wang X., Gocek E., Liu C.G., Studzinski G.P. (2009). MicroRNAs181 regulate the expression of p27Kip1 in human myeloid leukemia cells induced to differentiate by 1,25-dihydroxyvitamin D3. Cell Cycle.

[87] Bartel D.P. (2009). MicroRNAs: Target recognition and regulatory functions. Cell.

[88] Ji Y., Kutner A., Verstuyf A., Verlinden L., Studzinski G.P. (2002). Derivatives of vitamins D2 and D3 activate three MAPK pathways and upregulate pRb expression in differentiating HL60 cells. Cell Cycle.

[89] Eelen G., Verlinden L., Bouillon R., De Clercq P., Muñoz A., Verstuyf A. (2010). CD-ring modified vitamin D3 analogs and their superagonistic action. J. Steroid Biochem. Mol. Biol..

[90] Wilson A.J., Velcich A., Arango D., Kurland A.R., Shenoy S.M., Pezo R.C., Levsky J.M., Singer R.H., Augenlicht L.H. (2002). Novel detection and differential utilization of a c-myc transcriptional block in colon cancer chemoprevention. Cancer Res..

[91] Washington M.N., Weigel N.L. (2010). 1{alpha},25-Dihydroxyvitamin D3 inhibits growth of VCaP prostate cancer cells despite inducing the growth-promoting TMPRSS2:ERG gene fusion. Endocrinology.

[92] Palmer H.G., Gonzalez-Sancho J.M., Espada J., Berciano M.T., Puig I., Baulida J., Quintanilla M., Cano A., de Herreros A.G., Lafarga M. (2001). Vitamin D(3) promotes the differentiation of colon carcinoma cells by the induction of E-cadherin and the inhibition of beta-catenin signaling. J. Cell Biol..

[93] Studzinski G.P., Rathod B., Rao J., Kheir A., Wajchman H.J., Zhang F., Finan J.B., Nowell P.C. (1996). Transition to tetraploidy in 1,25-dihydroxyvitamin D3-resistant HL60 cells is preceded by reduced growth factor dependence and constitutive up-regulation of Sp1 and AP-1 transcription factors. Cancer Res..

[94] Godyn J.J., Xu H., Zhang F., Kolla S., Studzinski G.P. (1994). A dual block to cell cycle progression in HL60 cells exposed to analogues of vitamin D. Cell Prolif..

[95] Jiang F., Li P., Fornace A.J., Nicosia S.V., Bai W. (2003). G2/M arrest by 1,25-dihydroxyvitamin D3 in ovarian cancer cells mediated through the induction of GADD45 via an exonic enhancer. J. Biol. Chem..

[96] Fetahu I.S., Höbaus J., Kállay E. (2014). Vitamin D and the epigenome. Front. Physiol..

[97] Marik R., Fackler M., Gabrielson E., Zeiger M.A., Sukumar S., Stearns V., Umbricht C.B. (2010). DNA methylation-related vitamin D receptor insensitivity in breast cancer. Cancer Biol. Ther..

[98] Habano W., Gamo T., Terashima J., Sugai T., Otsuka K., Wakabayashi G., Ozawa S. (2011). Involvement of promoter methylation in the regulation of Pregnane X receptor in colon cancer cells. BMC Cancer.

[99] Höbaus J., Fetahu I.S., Khorchide M., Manhardt T., Kallay E. (2013). Epigenetic regulation of the 1,25-dihydroxyvitamin D3 24-hydroxylase (CYP24A1) in colon cancer cells. J. Steroid Biochem. Mol. Biol..

[100] Yamshchikov A., Desai N., Blumberg H., Ziegler T., Tangpricha V. (2009). Vitamin D for Treatment and Prevention of Infectious Diseases: A Systematic Review of Randomized Controlled Trials. Endocr. Pract..

[101] Chandel N., Husain M., Goel H., Salhan D., Lan X., Malhotra A., McGowan J., Singhal P.C. (2013). VDR hypermethylation and HIV-induced T cell loss. J. Leukoc. Biol..

[102] Novakovic B., Sibson M., Ng H.K., Manuelpillai U., Rakyan V., Down T., Beck S., Fournier T., Evain-Brion D., Dimitriadis E. (2009). Placenta-specific methylation of the gitamin D 24-gydroxylase gene implications for feedback autoregulation of active vitamin d levels at the fetomaternal interface. J. Biol. Chem..

[103] Khorchide M., Lechner D., Cross H.S. (2005). Epigenetic regulation of Vitamin D hydroxylase expression and activity in normal and malignant human prostate cells. J. Steroid Biochem. Mol. Biol..

[104] Luo W., Karpf A.R., Deeb K.K., Muindi J.R., Morrison C.D., Johnson C.S., Trump D.L. (2010). Epigenetic Regulation of Vitamin D 24-Hydroxylase/CYP24A1 in Human Prostate Cancer. Cancer Res..

[105] Johnson C.S., Chung I., Trump D.L. (2010). Epigenetic silencing of CYP24 in the tumor microenvironment. J. Steroid Biochem. Mol. Biol..

[106] Höbaus J., Hummel D.M., Thiem U., Fetahu I.S., Aggarwal A., Müllauer L., Heller G., Egger G., Mesteri I., Baumgartner-Parzer S. (2013). Increased copy-number and not DNA hypomethylation causes overexpression of the candidate proto-oncogene CYP24A1 in colorectal cancer. Int. J. Cancer.

